# Supplementary material for: Assessing the Impact of Retreat Mechanisms in a Simple Antarctic Ice Sheet Model Using Bayesian Calibration
Source: PLoS One. 2017 Jan 12;12(1):e0170052. doi: 10.1371/journal.pone.0170052 (PMC5231269; doi:10.1371/journal.pone.0170052)
Supplement: S1 Text — (PDF) [file pone.0170052.s001.pdf]

Supplementary material for:

Assessing the impact of retreat mechanisms in a simple Antarctic ice sheet model using Bayesian calibration

Kelsey L. Ruckert[1], Gary Shaffer[2,3], David Pollard[1], Yawen Guan[4], Tony E. Wong[1], Chris E. Forest[1,5,6], Klaus Keller[1,6,7,*]

1 Earth and Environmental Systems Institute, The Pennsylvania State University, University Park, Pennsylvania, USA
2 GAIA Antarctica, University of Magallanes, Punta Arenas, Chile
3 Niels Bohr Institute, University of Copenhagen, Copenhagen, Denmark
4 Department of Statistics, The Pennsylvania State University, University Park, Pennsylvania, USA
5 Department of Meteorology, The Pennsylvania State University, University Park, Pennsylvania, USA
6 Department of Geosciences, The Pennsylvania State University, University Park, Pennsylvania, USA
7 Department of Engineering and Public Policy, Carnegie Mellon University, Pittsburgh, Pennsylvania, USA

* Email: klaus@psu.edu

Discussion of constraints for DAIS calibration with a traceable account

Observational constraints

The DAIS (Danish Center for Earth System Science Antarctic Ice Sheet) model [1] is calibrated using four constraints over the last two glacial cycles. The constraints include the potential Antarctic ice sheet (AIS) contribution for the last interglacial (LIG, ∼120 ka BP), the last glacial maximum (LGM, ∼20 ka BP), the mid-Holocene (MH, ∼6 ka BP), and the instrumental period (∼2002). These constraints are from paleo-reconstructions and satellite techniques providing sea-level equivalent (SLE) estimates. The paleo-reconstructions are estimates based on various paleo-sea-level indicators including constructional coral terraces, coral microatolls, erosional features (wave-cut terraces, sea caves, bioerosional notches, raised beaches), microfossils, archaeological structures and artifacts, and geophysical models [2,3]. More importantly, regional and local responses (glacio/hydro-isostasy, tectonic movements, and deformations) are accounted for in these constraints.

Last interglacial

During the LIG, mean sea level was higher than present day, roughly 6.6–9.4 m higher [2]. The warmer temperatures during this time period likely caused thermal expansion of the ocean and melting of the glaciers and ice caps. However, this contribution was likely less than 1 m [1, 4]. The bulk of the rise in sea level is then estimated to come from the Greenland and Antarctic ice sheets. Multiple studies average a central range of 2.4–3.8 m contribution from the Greenland Ice Sheet [5–12]. After combining the contributions

from thermal expansion, glaciers and ice caps, and the Greenland ice sheet, 1.8–6.0 m remain. We assume this range to be explained by ice loss from the AIS and adopt the range for the LIG constraint.

Last glacial maximum

Over the past two decades multiple studies have estimated ranges of depression from the AIS in SLE from 2.37 m to 21 m during the LGM [13–23]. The most recent studies estimate a SLE depression less then 10 m [20–23]. Due to the deep uncertainty, we estimate a range by averaging the estimates with a greater than 10 m depression and adopt this as our lower bound and average the more recent estimates to use as our upper bound. Using this method we adopt the range of 6.9–15.8 m SLE depression from the AIS for the LGM constraint.

Mid-Holocene

During the MH, mean sea level was roughly 2–3 m below present day sea level [3]. However, temperatures were slightly warmer allowing for glaciers, ice caps, and the GIS to be potentially smaller than present day [24, 25]. It is possible that the AIS was larger in size to account for the warmer temperatures in addition to the lower global mean sea level. This implies a 2–4 m SLE depression from AIS during the MH [1]. However, other studies [18, 20], estimate a range of 0–1 m SLE depression from the AIS at 5 ka BP and 3.95 m at 6.8 ka BP, respectively. Taking these estimates into account we adopt a range of 1.25–4 m SLE depression for the MH constraint ($\sim$6 ka BP).

Instrumental Period

The age of satellite geodesy has since revolutionized estimating ice sheet mass balance. Since the 1990s, many studies estimate ice sheet mass balance using satellite techniques of altimetry, interferometry, and gravimetry [26]. A recent study [26] combines an ensemble of these datasets along with models of surface mass balance based on their common geographical regions and time intervals. Accounting for glacial isostatic adjustment, the authors estimate the rate of change in mass balance from 1992 to 2011 for the Antarctic Peninsula (-20 $\pm$ 14 Gt/yr), East Antarctica (14 $\pm$ 43 Gt/yr), West Antarctica (-65 $\pm$ 26 Gt/yr), and all of Antarctica (-71 $\pm$ 53 Gt/yr) [26]. The results are represented with a 1-σ error. Using the rate for the total AIS, we convert the measurement to SLE and accumulate to the year 2002. We hence adopt 2.0 $\pm$ 0.93 mm SLE (expanded to a 2-σ error and relative to the year 1992) as the instrumental period constraint.

It is important to note that these constraints are deeply uncertain and can be interpreted in many ways. More research is needed to better understand past sea level contributions from different sources (for example, the wide range of estimates for the LGM). For simplicity, we define estimates described as a range to be a 95% confidence interval or 2-σ error and estimates described as an error to be 1-σ errors, unless the probability of the estimates is clearly specified in the article. For this study, we treat our adopted ranges as 95% confidence intervals or 2-σ errors to account for any additional unresolved internal variability.

References

[1] Shaffer G. Formulation, calibration and validation of the DAIS model (version 1), a simple Antarctic ice sheet model sensitive to variations of sea level and ocean subsurface temperature. Geoscientific Model Development, 2014;7(4):1803–1818.

[2] Kopp RE, Simons FJ, Mitrovica JX, Maloof AC, and Oppenheimer M. Probabilistic assessment of sea level during the last interglacial stage. Nature, 2009;462:863–867.

[3] Lambeck K, Woodroffe CD, Antonioli F, Anzidei M, Gehrels WR, Laborel J, et al. Paleoenvironmental records, geophysical modeling, and reconstruction of sea-level trends and variability on centennial and longer timescales. In Understanding Sea-Level Rise and Variability, 2010;61–121. Wiley-Blackwell.

[4] Dutton A and Lambeck K. Ice volume and sea level during the last interglacial. Science, 2012;337(6091):216–219.

[5] Cuffey KM and Marshall SJ. Substantial contribution to sea-level rise during the last interglacial from the Greenland ice sheet. Nature, 2000;404:591–594.

[6] Tarasov L and Peltier WR. Greenland glacial history, borehole constraints, and Eemian extent. Journal of Geophysical Research: Solid Earth, 2003;108(B3):n/a–n/a. 2143.

[7] Lhomme N, Clarke GKC, and Marshall SJ. Tracer transport in the Greenland ice sheet: constraints on ice cores and glacial history. Quaternary Science Reviews, 2005;24(1–2):173–194.

[8] Otto-Bliesner BL, Marshall SJ, Overpeck JT, Miller GH, and Hu A. Simulating arctic climate warmth and icefield retreat in the last interglaciation. Science, 2006;311(5768):1751–1753.

[9] Alley RB, Andrews JT, Brigham-Grette J, Clarke GKC, Cuffey KM, Fitzpatrick JJ, et al. History of the Greenland ice sheet: paleoclimatic insights. Quaternary Science Reviews, 2010;29(15–16):1728–1756. Special Theme: Arctic Palaeoclimate Synthesis (PP. 1674-1790).

[10] Colville EJ, Carlson AE, Beard BL, Hatfield RG, Stoner JS, Alberto V. Reyes AV, et al. Sr-nd-pb isotope evidence for ice-sheet presence on southern Greenland during the last interglacial. Science, 2011;333(6042):620–623.

[11] Dahl-Jensen D, Albert MR, Aldahan A, Azuma N, Balslev-Clausen D, Baumgartner M, et al. Eemian interglacial reconstructed from a Greenland folded ice core. Nature, 2013;493:489–494.

[12] Stone EJ, Lunt DJ, Annan JD, and Hargreaves JC. Quantification of the Greenland ice sheet contribution to last interglacial sea level rise. Climate of the Past, 2013;9(2):621–639.

[13] Ritz C, Rommelaere V, and Dumas C. Modeling the evolution of Antarctic ice sheet over the last 420,000 years: Implications for altitude changes in the vostok region. Journal of Geophysical Research: Atmospheres, 2001;106(D23):31943–31964.

[14] Clark PU and Mix AC. Ice sheets and sea level of the last glacial maximum. Quaternary Science Reviews, 2002;21(1–3):1–7.

[15] Huybrechts P. Sea-level changes at the LGM from ice-dynamic reconstructions of the Greenland and Antarctic ice sheets during the glacial cycles. Quaternary Science Reviews, 2002;21(1–3):203–231.

[16] Peltier WR. On eustatic sea level history: Last glacial maximum to Holocene. Quaternary Science Reviews, 2002;21(1–3):377–396.

[17] Peltier WR. Global glacial isostasy and the surface of the ice-age earth: The ice-5g (vm2) model and grace. Annual Review of Earth and Planetary Sciences, 2004;32(1):111–149.

[18] Ivins ER and James TS. Antarctic glacial isostatic adjustment: a new assessment. Antarctic Science, 2005;17(4):541–553.

[19] Philippon G, Ramstein G, Charbit S, Kageyama M, Ritz C, and Dumas C. Evolution of the Antarctic ice sheet throughout the last deglaciation: A study with a new coupled climate—north and south hemisphere ice sheet model. Earth and Planetary Science Letters, 2006;248(3–4):750–758.

[20] Whitehouse PL, Bentley MJ, and Le Brocq AM. A deglacial model for Antarctica: geological constraints and glaciological modelling as a basis for a new model of Antarctic glacial isostatic adjustment. Quaternary Science Reviews, 2012;32:1–24.

[21] Golledge NR, Fogwill CJ, Mackintosh AN, and Buckley KM. Dynamics of the last glacial maximum Antarctic ice-sheet and its response to ocean forcing. Proceedings of the National Academy of Sciences, 2012;109(40):16052–16056.

[22] Gomez N, Pollard D, and Mitrovica JX. A 3-d coupled ice sheet – sea level model applied to Antarctica through the last 40 ky. Earth and Planetary Science Letters, 2013;384:88–99.

[23] Pollard D, Chang W, Haran M, Applegate P, and DeConto R. Large ensemble modeling of the last deglacial retreat of the west Antarctic ice sheet: comparison of simple and advanced statistical techniques. Geoscientific Model Development, 2016;9(5):1697–1723.

[24] Vinther BM, Buchardt SL, Clausen HB, Dahl-Jensen D, Johnsen SJ, Fisher DA, et al. Holocene thinning of the Greenland ice sheet. Nature, 2009;461:385–388.

[25] Marcott SA, Clark PU, Padman L, Klinkhammer GP, Springer SR, Liu Z, et al. Ice-shelf collapse from subsurface warming as a trigger for heinrich events. Proceedings of the National Academy of Sciences, 2011;108(33):13415–13419.

[26] Shepherd A, Ivins ER, A G, Barletta VR, Bentley MJ, Bettadpur S, et al. A reconciled estimate of ice-sheet mass balance. Science, 2012;338(6111):1183–1189.
