# Supplementary material for: Assessing the Impact of Retreat Mechanisms in a Simple Antarctic Ice Sheet Model Using Bayesian Calibration
Source: PLoS One. 2017 Jan 12;12(1):e0170052. doi: 10.1371/journal.pone.0170052 (PMC5231269; doi:10.1371/journal.pone.0170052)
Supplement: S1 Table — A traceable account of previous studies used to create the constraints is listed in the references. (PDF) [file pone.0170052.s002.pdf]

Time	Range [m]	Median estimate [m]	Error [m]	References [m]
Instrumental period (1992-2011 [2002])	0.001 - 0.0029	0.0020	0.00093***	**Estimated AIS contribution:**<br>Shepherd et al. (2012) -71 +/- 53 Gt/yr **
Mid-Holocence (~6kyr BP)	-1.25 - -4	-2.6	+/- 1.4***	Vinther (2009); Marcott et al. (2013);<br>**Global eustatic sea-level rise:**<br>Lambeck (2010) 2-3*** below today;<br>**Estimated AIS contribution:**<br>Ivins & James (2005) [6.8ka bp] 3.95*;<br>Whitehouse (2012) [5ka bp] 0.5 +/- 0.5***;<br>Shaffer (2014) 2-4***
Last glacial maximum (~20kyr BP)	-6.9 - -15.8	-11.4	+/- 4.5***	**Estimated AIS contribution:**<br>Ritz et al. (2001) 2.37 - 5.91***;<br>Clark & Mix (2002) 14 – 21***;<br>Huybrechts (2002) 13 - 21***, 14 - 18**, 17.5*;<br>Peltier (2002) 17.3* & 17.6* [+ Patagonia];<br>Peltier (2004) 17.3*;<br>Ivins & James (2005) 10.12*;<br>Whitehouse (2012) 9 +/- 1.5***, 8*;<br>Golledge et al. (2013) 6.67*;<br>Gomez et al. (2013) 5 – 9.5***;<br>Pollard et al. (2016) 5 – 10**
Last interglacial (~120kyr BP)	1.8 – 6.0	3.9	+/-2.1***	**Global eustatic sea level rise:**<br>Kopp et al. (2009) 6.6 - 9.4***;<br>**Ocean warming & Glacier/ice cap melt:**<br>Dutton & Lambeck (2012) & Shaffer (2014) ≤1.0***;<br>**Estimated GIS contribution:**<br>Cuffey & Marshall (2000) 4 - 5.5***;<br>Tarasov & Peltier (2003) 2 - 5.2***, 2.7 - 4.5**;<br>Lhomme et al. (2005) 3.5 - 4.5***;<br>Otto-Bliesner et al. (2006) 2.2 - 3.4***;<br>Ally et al. (2010) 3 – 4***;<br>Colville et al. (2011) 1.6 - 2.2***;<br>Dahl-Jensen et al. (2013) 2*;<br>Stone et al. (2013) 0.4 – 3.8***
*Best estimate	**error(1-sigma)	***range (95%, 2-sigma)		
