# Supplementary material for: Assessing the Impact of Retreat Mechanisms in a Simple Antarctic Ice Sheet Model Using Bayesian Calibration
Source: PLoS One. 2017 Jan 12;12(1):e0170052. doi: 10.1371/journal.pone.0170052 (PMC5231269; doi:10.1371/journal.pone.0170052)
Supplement: S2 Table — This table was accessed and modified on 13 June 2016 from http://cmip-pcmdi.llnl.gov/cmip5/docs/CMIP5_modeling_groups.docx. (PDF) [file pone.0170052.s003.pdf]

**Modeling Center (or Group)**	**Institute ID**	**Model Name**
Centre National de Recherches Météorologiques / Centre Européen de Recherche et Formation Avancée en Calcul Scientifique	CNRM-CERFACS	CNRM-CM5
