# Supplementary figures and images for: Assessing the Impact of Retreat Mechanisms in a Simple Antarctic Ice Sheet Model Using Bayesian Calibration
Source: PLoS One. 2017 Jan 12;12(1):e0170052. doi: 10.1371/journal.pone.0170052 (PMC5231269; doi:10.1371/journal.pone.0170052)

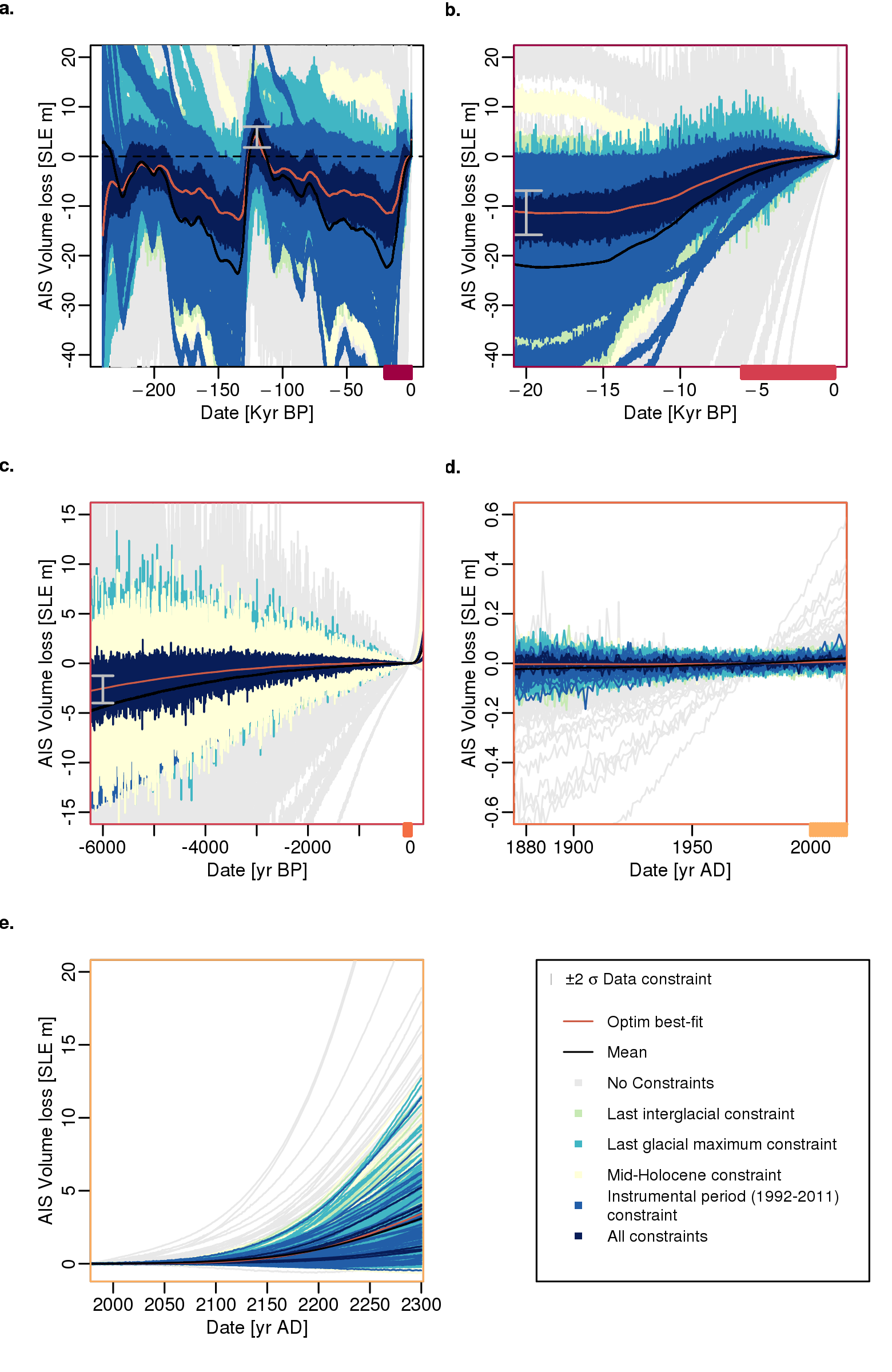

Supplement: S1 Fig — Shown are the realizations pre-calibrated with the Last interglacial constraint (green), Last glacial maximum constraint (turquoise), mid Holocene constraint (yellow), instrumental period constraint (blue), all the constraints (navy), and no constraints (gray). The brown line represented the optimized fit and the bars are the observational constraints. (TIFF) [file pone.0170052.s004.tiff]

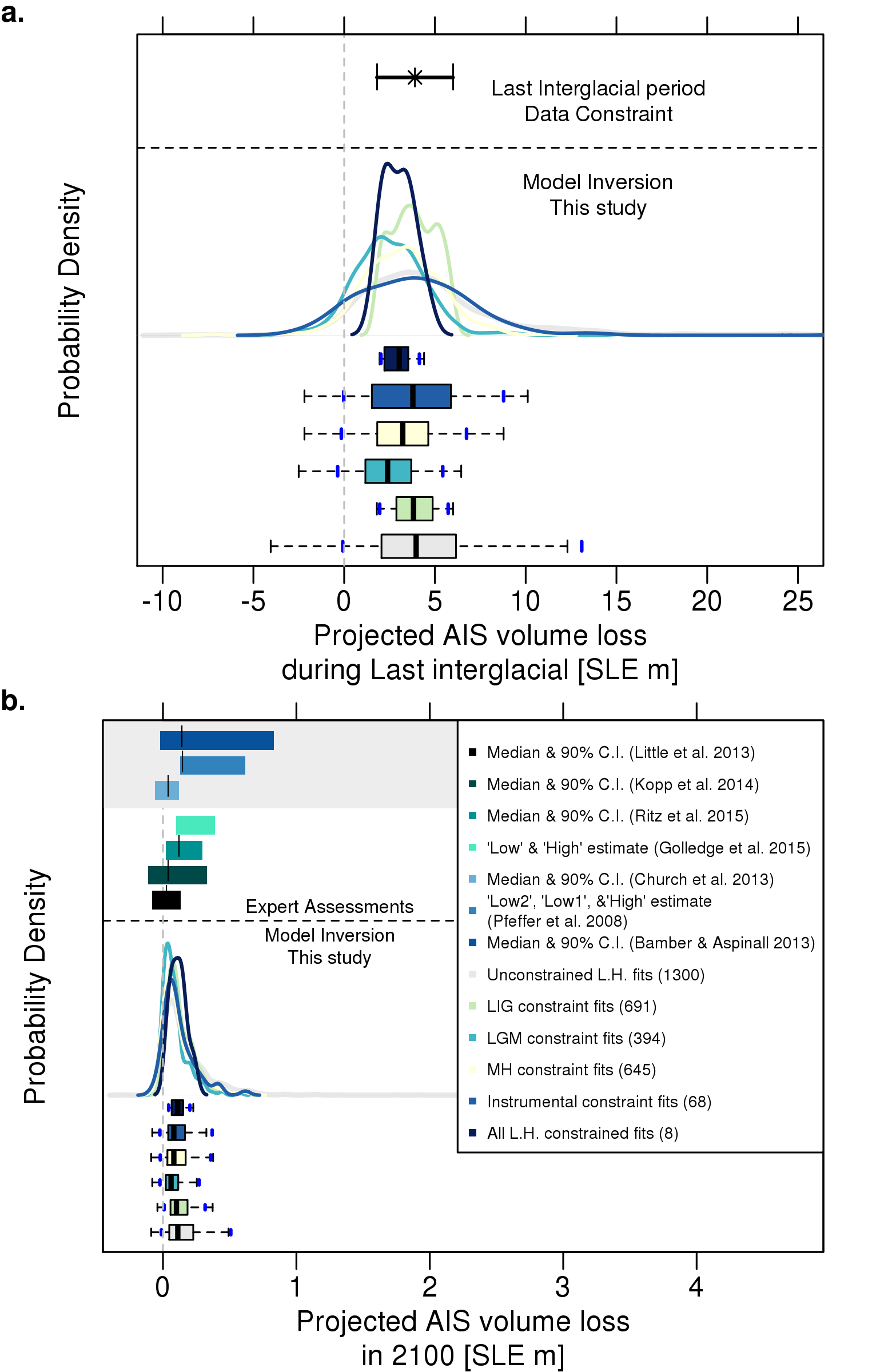

Supplement: S2 Fig — The different coloured probability density functions represent realizations using different constraints (last interglacial (green), last glacial maximum (turquoise), mid-Holocene (yellow), instrumental period (blue), all the constraints (navy), and no constraints (gray)). The black range represents the reconstructed AIS contribution to global sea level during the last interglacial period. The different coloured lines are the 90% confidence/credible intervals with their estimated medians (∣) from previous AIS studies. The bars in shades of green are from model assessments projected with the RCP 8.5 or similar scenario. Bars in shades of blue, highlighted in grey, are from non-model assessments. (PNG) [file pone.0170052.s005.png]

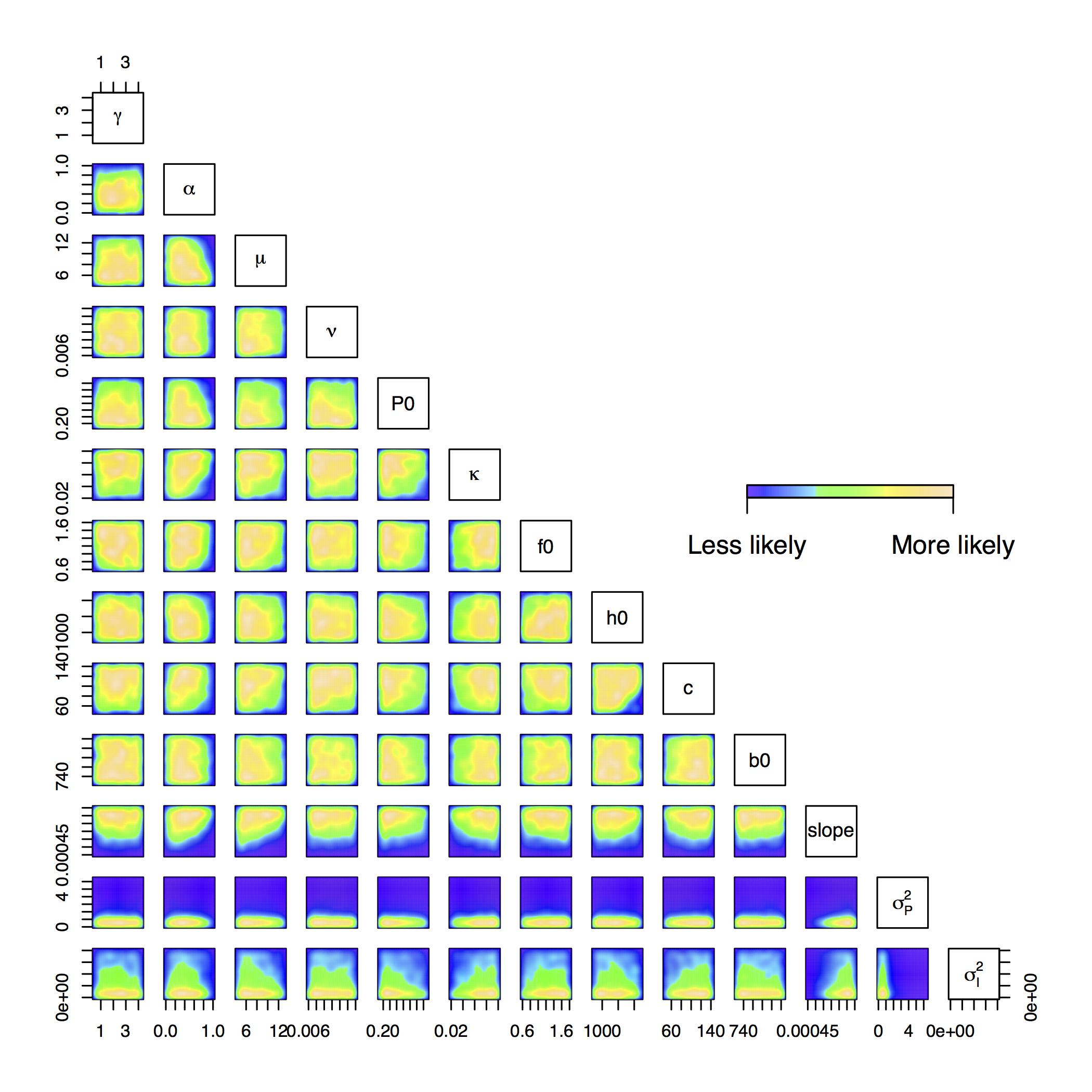

Supplement: S4 Fig — Values increase in likelihood from blue to tan. (PNG) [file pone.0170052.s007.png]
