# Supplementary material for: Assessing the Impact of Retreat Mechanisms in a Simple Antarctic Ice Sheet Model Using Bayesian Calibration
Source: PLoS One. 2017 Jan 12;12(1):e0170052. doi: 10.1371/journal.pone.0170052 (PMC5231269; doi:10.1371/journal.pone.0170052)

Parameter	Description	Units	Min.	Max.
ϒ	Power for the relation of ice flow speed to water depth		0.5	4.25
α	Partition parameter for effect of ocean subsurface temp. in ice flux		0	1
μ	Profile parameter for parabolic ice sheet surface	m^1/2	4.35	13.05
ν	Proportionality constant relating the runoff decrease with height to precip.	m^-1/2 yr^-1/2	6 x 10^-3	0.018
P0	Annual precip. for air temp. equal to 0 °C	m of ice/yr	0.175	0.525
κ	Coefficient for the exponential dependency of precip. on air temp.	°C^-1	0.02	0.06
*f*0	Proportionality constant relating the runoff decrease with height to precip.	m/yr	0.6	1.8
h0	Runoff line height for mean Antarctic temp. reduced to sea-level equal to 0 °C	m	735.5	2206.5
c	Proportionality constant for the dependency of runoff line height on air temp.	m [°C]^-1	47.5	142.5
b0	Undisturbed bed height at the continent center	m	725	825
slope	Slope of the undisturbed bed		4.5 x 10^-4	7.5 x 10^-4
σ^2_P	Bias from paleo-observation error and unresolved internal variability/ model error		0	-
σ^2_I	Bias from instrumental period observation error and unresolved internal variability/ model error		0	4.0 x 10^-4

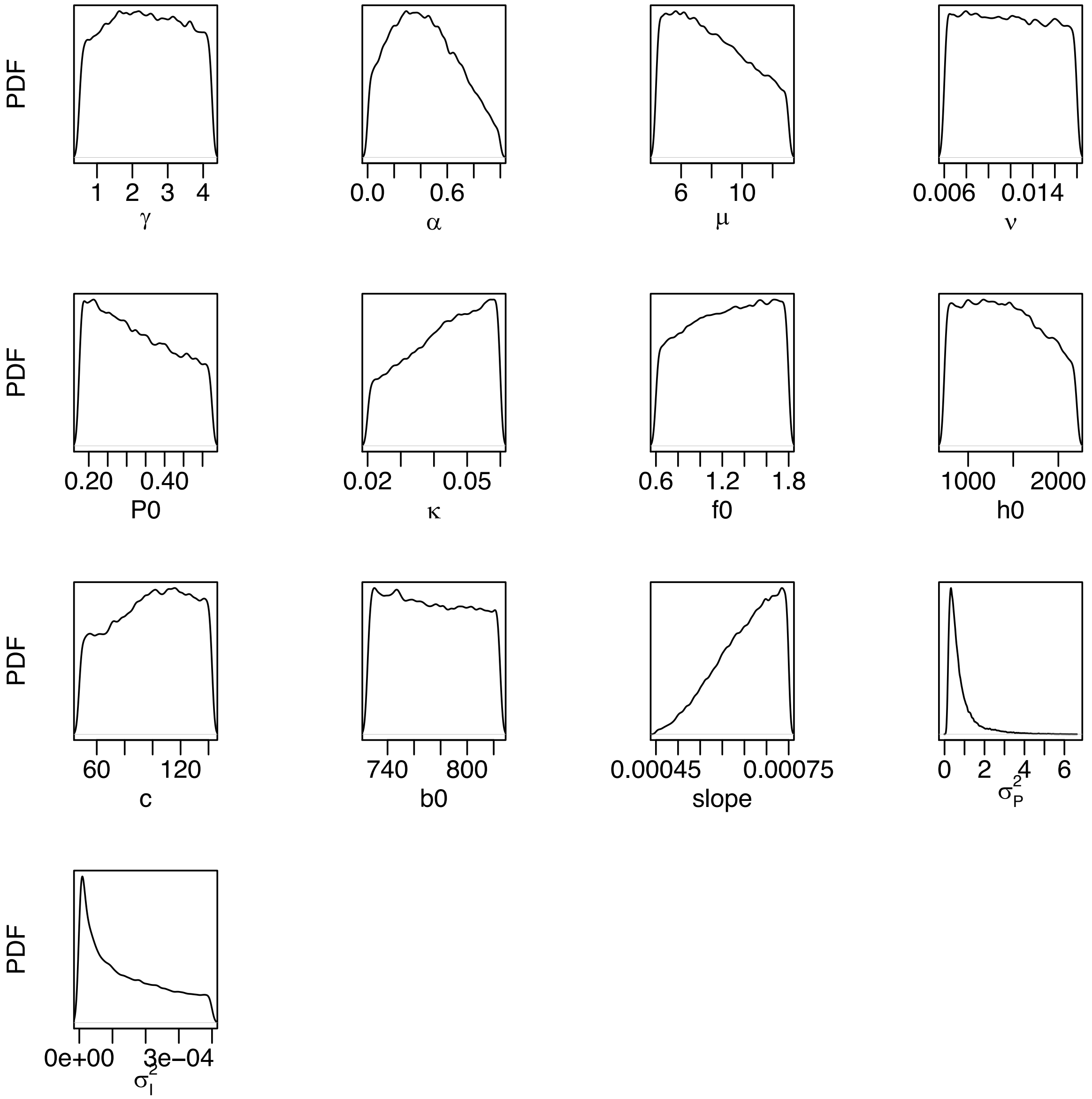

Supplement: S3 Fig — The horizontal axis range represents the lower and upper bounds of the uniform prior distributions. The variance parameter σ2P uses an inverse gamma prior distribution (α = 2, β = 1) with a minimum value of 0 and an infinite upper bound. The table indicates the estimated parameters their prior ranges. (PDF) [file pone.0170052.s006.pdf]
